# Comparison of risk classification between EndoPredict and MammaPrint in ER-positive/HER2-negative primary invasive breast cancer

**DOI:** 10.1371/journal.pone.0183452

**Published:** 2017-09-08

**Authors:** Alberto Peláez-García, Laura Yébenes, Alberto Berjón, Antonia Angulo, Pilar Zamora, José Ignacio Sánchez-Méndez, Enrique Espinosa, Andrés Redondo, Victoria Heredia-Soto, Marta Mendiola, Jaime Feliú, David Hardisson

**Affiliations:** 1 Department of Pathology, Hospital Universitario La Paz, IdiPAZ, Madrid, Spain; 2 Molecular Pathology and Therapeutic Targets Group, Hospital Universitario La Paz, IdiPAZ, Madrid, Spain; 3 Molecular Pathology Diagnostic Unit, Hospital Universitario La Paz, INGEMM, IdiPAZ, Madrid, Spain; 4 Myriad Genetics España SLU, Madrid, Spain; 5 Department of Medical Oncology, Hospital Universitario La Paz, IdiPAZ, Madrid, Spain; 6 Faculty of Medicine, Universidad Autónoma de Madrid, Madrid, Spain; 7 Translational Oncology Group, Hospital Universitario La Paz, IdiPAZ, Madrid, Spain; 8 Department of Obstetrics and Gynecology, Breast Cancer Unit, Hospital Universitario La Paz, IdiPAZ, Madrid, Spain; 9 Centro de Investigación Biomédica en Red de Cáncer (CIBERONC), Instituto de Salud Carlos III, Ministerio de Economía, Industria y Competitividad, Madrid, Spain; University of North Carolina at Chapel Hill School of Medicine, UNITED STATES

## Abstract

**Purpose:**

To compare the concordance in risk classification between the EndoPredict and the MammaPrint scores obtained for the same cancer samples on 40 estrogen-receptor positive/HER2-negative breast carcinomas.

**Methods:**

Formalin-fixed, paraffin-embedded invasive breast carcinoma tissues that were previously analyzed with MammaPrint as part of routine care of the patients, and were classified as high-risk (20 patients) and low-risk (20 patients), were selected to be analyzed by the EndoPredict assay, a second generation gene expression test that combines expression of 8 genes (EP score) with two clinicopathological variables (tumor size and nodal status, EPclin score).

**Results:**

The EP score classified 15 patients as low-risk and 25 patients as high-risk. EPclin re-classified 5 of the 25 EP high-risk patients into low-risk, resulting in a total of 20 high-risk and 20 low-risk tumors. EP score and MammaPrint score were significantly correlated (p = 0.008). Twelve of 20 samples classified as low-risk by MammaPrint were also low-risk by EP score (60%). 17 of 20 MammaPrint high-risk tumors were also high-risk by EP score. The overall concordance between EP score and MammaPrint was 72.5% (κ = 0.45, (95% CI, 0.182 to 0.718)). EPclin score also correlated with MammaPrint results (p = 0.004). Discrepancies between both tests occurred in 10 cases: 5 MammaPrint low-risk patients were classified as EPclin high-risk and 5 high-risk MammaPrint were classified as low-risk by EPclin and overall concordance of 75% (κ = 0.5, (95% CI, 0.232 to 0.768)).

**Conclusions:**

This pilot study demonstrates a limited concordance between MammaPrint and EndoPredict. Differences in results could be explained by the inclusion of different gene sets in each platform, the use of different methodology, and the inclusion of clinicopathological parameters, such as tumor size and nodal status, in the EndoPredict test.

## Introduction

The decision on adjuvant treatment for breast cancer patients is based on risk assessment using clinicopathological criteria, such as patient age, menopausal status, axillary lymph node status, tumor size, tumor grade, estrogen receptor (ER)/progesterone receptor (PgR) expression, HER2 status, and Ki67 score. However, adjuvant treatment decision making in women with ER+/HER2- early breast cancer remains as a difficult task. Routinely, all these patients will receive adjuvant hormonal treatment. A substantial proportion of these patients are also treated with adjuvant chemotherapy, although a significant part of these will not benefit from this treatment [1]. Thus, a major challenge for clinical oncologists is to identify those patients who will not benefit for adjuvant chemotherapy, and those who are more likely to develop recurrence, so that the most appropriate therapeutic regime can be administered in each case [1,2].

In recent years, molecular characterization of breast cancer has contributed to improving our understanding of breast cancer as a complex disease, and led to the development of a variety of prognostic and predictive gene signatures that may also be useful in recurrence prediction and treatment decision making [3]. These molecular tests provide useful prognostic and predictive information that is independent of standard clinicopathological information, and may be particularly helpful in cases for which measures of clinical risk are equivocal (i.e., small, node-negative, intermediate grade tumors) [4]. However, these genomic tests are based in different methodology and measure different genes. Moreover, they have been validated clinically in different cohorts from randomized clinical trials with long-term clinical outcomes. Recent studies highlight the functional and clinically relevant impact of these multigene prognostic assays upon the adjuvant chemotherapy in ER+ early-stage breast carcinoma, indicating chemotherapy changes in approximately 25–30% of patients, with more changes against than for adjuvant chemotherapy [5].

One of the most widely used test is the MammaPrint (MP) assay (Agendia Laboratories, Amsterdam, The Netherlands), which is a prognostic score performed by a central laboratory that was cleared by the FDA in 2007. MP was initially limited by its requirement for fresh tissue, but it is now validated for formalin-fixed, paraffin-embedded (FFPE) tissue [6]. MP measures the expression of 70 genes using a microarray platform, and reports a binary risk classification (low-risk or high-risk) for recurrence without adjuvant chemotherapy. This information is intended to spare patients at low-risk of recurrence from receiving adjuvant chemotherapy, with its attendant morbidity [7]. More recently developed, the EndoPredict assay (EP) (Sividon Diagnostics GmbH, Cologne, Germany), is a test based on gene expression data in combination with two clinicopathological risk parameters (tumor and nodal status) to assess the risk of distant metastasis in patients with ER+/HER2- primary breast cancer if treated with adjuvant endocrine therapy alone [8]. This test measures the expression of eight cancer-related genes of interest (*BIRC5*, *UBE2C*, *DHCR7*, *RBBP8*, *IL6ST*, *AZGP1*, *MGP* and *STC2*) and three reference genes (*CALM2*, *OAZ1* and *RPL37A*) to calculate a molecular risk score (EP score). The molecular risk score is then combined with the nodal status and tumor size resulting in a molecular-clinicopathological hybrid score (EPclin score) with improved prognostic power. Using a predefined cutoff value, patients are stratified into low- or high-risk of distant recurrence. The test can be carried out on routinely processed and archived FFPE tissue, and is designed to be performed decentrally [9,10]. EP was validated in three randomized phase III trials with patients with ER+/HER2-, node-negative and node-positive breast carcinomas [8,11]. The EP provided additional prognostic information to conventional risk factors such as grading, quantitative ER, or Ki67 and outperformed risk classification by clinical guidelines. Moreover, it could be demonstrated that EP is prognostic for early and late metastasis [11,12]. The EPclin score was also directly compared to purely clinical risk classifications (like St. Gallen, German S3, and NCCN) and found to be superior to these classifiers [12].

The main objective of this study was to compare retrospectively the concordance in risk classification between the EndoPredict and the MammaPrint tests in 40 ER+/HER2- breast carcinomas. We further compared TargetPrint (Agendia Laboratories), a commercially available mRNA-based gene expression test that quantitatively determines gene expression levels of ER, PgR, and HER2 with conventional immunohistochemistry/FISH analysis of ER, PgR, and HER2.

## Materials and methods

### Ethical standards

The study was approved by the Ethics Committee of the University Hospital La Paz, Madrid, Spain (code HULP: PI-2146). Patients provided written consent for their samples to be used in this study.

### Patients and tumor samples

This study involved 40 patients with ER+/HER2- breast carcinomas tests which were previously categorized as low-risk (20 patients) or high-risk (20 patients) with MammaPrint as part of routine care of the patients. The main criterion for the selection of the patients was the result of the MammaPrint test (low-risk *versus* high-risk). All patients underwent surgery between March 2012 and December 2015 at the University Hospital La Paz, Madrid, Spain. Data on age and tumor characteristics were collected for all patients. The surgical specimens were fixed in 10% buffered formalin and embedded in paraffin. Four-μm thick sections were stained with hematoxylin-eosin for histological diagnosis. Sections (10μm) with at least 40% of tumor cellularity were selected for the study.

### Immunohistochemistry for ER/PgR/HER2 and Ki67 and fluorescence in situ hybridization (FISH) for HER2

All cases were reviewed by two breast pathologists (DH and LY) to assess tumor grade (using the Nottingham histological three-tier grading system), tumor size, nodal status, ER, PgR, HER2, and Ki67 expression. The expression of ERα (clone EP1; Dako, Glostrup, Denmark, prediluted), PgR (clone PgR1294; Dako, prediluted), and Ki67 (clone MIB1; Dako, prediluted) were determined by immunohistochemistry (IHC) during routine pathologic examination. ER and PgR status was determined based on the percentage of positive nuclei in the invasive neoplastic compartment of the tissue. Tumors were classified as ER- or PgR-positive when ≥1% invasive tumor cells showed definite nuclear staining, regardless of staining intensity. Ki67 was evaluated as the percentage of positively stained nuclear cancer cells (regardless of staining intensity). HER2 expression was evaluated with the HercepTest kit (Dako) and scored as 0, 1+, 2+, or 3+, according to the ASCO-CAP guidelines. Tumors scored as 2+ were re-tested with FISH using the HER2 IQFISH PharmDx kit (Dako).

### Mammaprint test

The MammaPrint test was performed on representative paraffin blocks of the breast carcinomas at the centralized Agendia Laboratories (Amsterdam, The Netherlands) blinded for clinical and histological data as part of routine care of the patients included in this study. TargetPrint assay was also performed in these cases.

### EndoPredict test

The same tumor tissue block used for MammaPrint testing in each patient was used for EP test. RNA extraction was performed as previously described [9]. Total RNA was extracted from one 10-μm whole FFPE tissue section using a silica-coated magnetic bead-based method with Tissue Preparation Reagents (Sividon Diagnostics). Expression of eight genes of interest (*AZGP1*, *BIRC5*, *DHCR7*, *IL6ST*, *MGP*, *RBBP8*, *STC2*, *UBE2C*), three normalization genes (*CALM2*, *OAZ1*, *RPL37A*) as well as the amount of residual genomic DNA (*HBB*) were assessed by the EP assay (Sividon Diagnostics). Gene expression was analyzed by one-step RT-qPCR using the SuperScript III PLATINUM One-Step Quantitative RT-PCR System with ROX (Invitrogen, Karlsruhe, Germany) according to manufacturer’s instructions in a VERSANT^®^ kPCR Molecular System (Siemens Healthcare Diagnostics, Erlangen, Germany). EP and EPclin scores were determined as published earlier [8,9] using the EndoPredict Report Generator software which is available online (www1.endopredict.com). The predefined cutoffs for diagnostic decisions were applied to stratify patients into low- or high-risk groups: EP low-risk (<5), EP high-risk (≥5); EPclin low-risk (<3.3), EPclin high-risk (≥3.3). The EPclin cut off value corresponds to a 10% distant recurrence rate at 10 years.

### Statistical analyses

The concordance between EP and MP was analyzed using Cohen's kappa and Fisher’s exact test. The association between the clinicopathological features and EP and EPclin scores was analyzed using the Pearson correlation or Fisher’s exact test, as appropriate. The correlation between the Ki67 and EP and EPclin scores was examined using the Pearson correlation coefficient. Agreement measurements between binary (positive *versus* negative) TargetPrint mRNA and IHC classifications were based on two-way contingency table analysis and included overall correlation and positive agreement (defined as the number of samples classified positive by both IHC and mRNA divided by the number of positive samples using IHC) [13]. Statistical analysis was performed with the SPSS statistics 19 software (IBM, Armonk, NY, USA).

## Results

### Patient characteristics

The characteristics of the 40 patients included in this study are summarized in Table 1. The detailed clinical, pathological, immunohistochemical and molecular data of the patients are shown in S1 Table.

**Table 1:** Characteristics of the 40 patients included in the study.

Characteristics	Patients N = 40 (%)
**Age at diagnosis (years)**	
≤55	17 (42.5)
>55	23 (57.5)
**Histology**	
Ductal	34 (85)
Lobular	6 (15)
**Tumor grade**	
1	8 (20)
2	22 (55)
3	10 (25)
**Tumor size**	
pT1b (0.5 to 1 cm)	9 (22.5)
pT1c (>1 to 2 cm)	22 (55)
pT2 (>2 to 5 cm)	9 (22.5)
**Nodal status**	
Negative	32 (80)
Positive*	8 (20)
**ER status**	
Positive	40 (100)
Negative	0 (0)
**PgR status**	
Positive	38 (95)
Negative	2 (5)
**HER2 status**	
Positive	0 (0)
Negative	40 (100)

ER, estrogen receptor; PgR, progesterone receptor;

*All micrometastases (pN1mi)

### MammaPrint test

MammaPrint test classified the breast carcinomas as low-risk in 20 patients and high-risk in 20 patients.

### EndoPredict test

According to the molecular EP score 15 patients were classified as low-risk and 25 patients as high-risk. The EPclin score (combining EP score with tumor size and nodal status) re-classified 5 of the 25 EP high-risk patients into the low-risk group resulting in 20 patients with low- and 20 patients with high-risk of distant recurrence. The clinicopathological characteristics of the patients according to EPclin score are summarized in Table 2.

**Table 2:** Clinical characteristics of the patients classified as high- or low-risk for distant metastasis by EPclin score.

	EndoPredict EPclin Low-risk n = 20	EndoPredict EPclin High-risk n = 20	P-value
**Age (years)**			
≤55	8	9	0.784
>55	12	11	
**Histology**			
Ductal	16	18	0.652
Lobular	4	2	0.292
**Tumor grade**			
1	5	3	0.429
2	13	9	0.204
3	2	8	0.028
**Tumor size**			
pT1b	5	4	0.705
pT1c	13	9	0.204
pT2	2	7	0.050
**Nodal status**			
Positive*	3	5	0.625
Negative	17	15	

*All micrometastases (pN1mi)

### Correlation and concordance between EP scores (EP molecular score and EPclin score) and MammaPrint test results

EP molecular scores and MP scores were significantly correlated (p = 0.008). Twelve of 20 samples classified as low-risk by MP were also low-risk by EP score (60%). Seventeen of 20 MP high-risk samples were also EP high-risk (85%). The overall concordance between both risk classifications was 72.5% (Table 3). Cohen’s κ was calculated to determine the agreement between EP and MP in risk stratification. There was a moderate agreement between the two-molecular test, κ = 0.45 (95% CI, 0.182 to 0.718), p < .0005.

**Table 3:** Comparison of EPscore and MammaPrint based risk classification.

		**EP score**	
	**n = 40**	**Low-risk**	**High-risk**
**Mammaprint**	**Low-risk**	12 (60%)	8 (40%)
	**High-risk**	3 (15%)	17 (85%)

The EPclin score also correlated with the MP results (p = 0.004). Fifteen of 20 samples classified as low-risk by MP were also low-risk by EPclin score (75%). Similarly, 15 of 20 MP high-risk patients were EPclin high-risk (75%). Overall concordance was 75% (Table 4). Cohen's κ between EPclin and MP was κ = 0.5 (95% CI, 0.232 to 0.768), p < .0005.

**Table 4:** Comparison of EPclin score and MammaPrint based risk classification.

		**EPclin score**	
	**n = 40**	**Low-risk**	**High-risk**
**Mammaprint**	**LowRisk**	15 (75%)	5 (25%)
	**High Risk**	5 (255%)	15 (75%)

### Correlation of proliferation index Ki67 to EP molecular and EPclin scores

**Fig 1:**
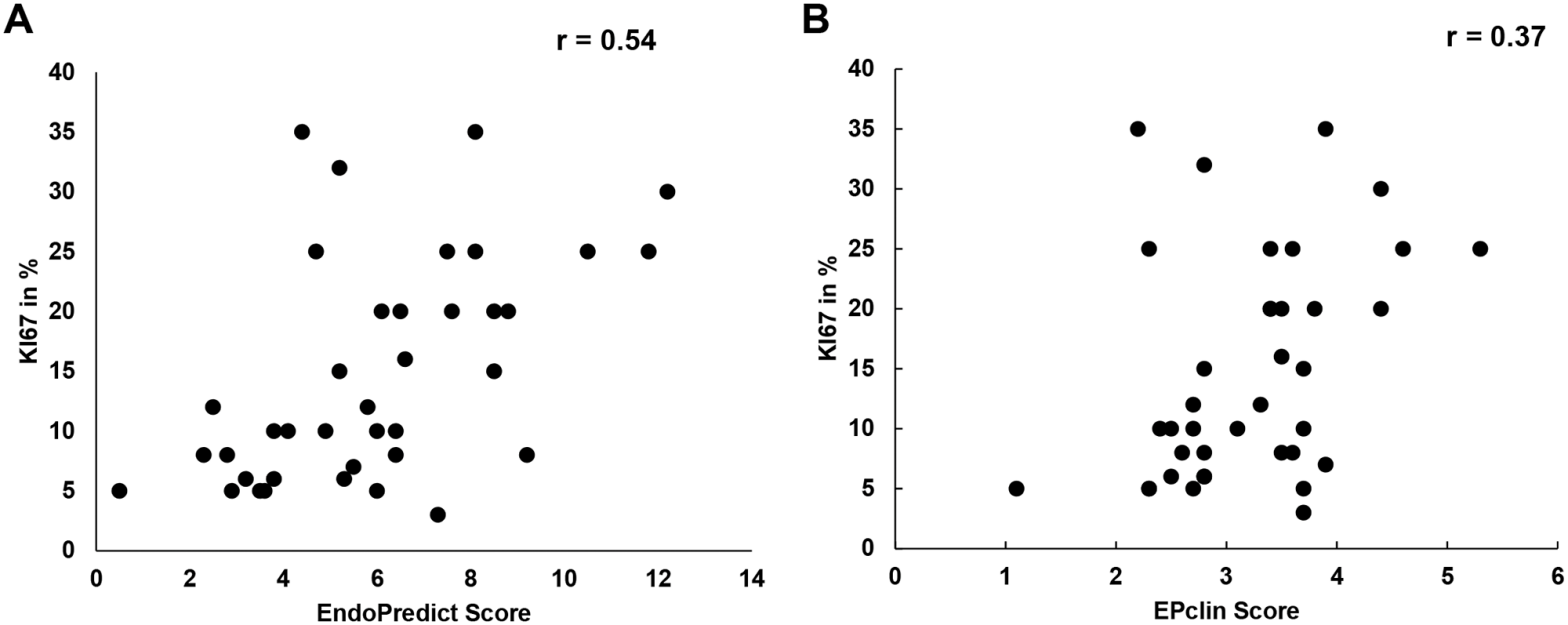
Comparison of proliferative index (Ki67) with EP score (A) and EPclin score (B). r = Pearson coefficient.

There was a statistically significant but moderate correlation between the EP score and Ki67 (Pearson coefficient = 0.535, p = 0.01) (Fig 1A). No significant correlation was found between EPclin score and Ki67 proliferation index (Pearson coefficient = 0.37, p = 0.05) (Fig 1B).

### Comparison of ER/PgR/HER2 status with conventional IHC/FISH and TargetPrint

Qualitative IHC/FISH (positive *versus* negative) showed a high concordance with TargetPrint readout (Table 5): of the 40 ER-positive cases by IHC, 39 (97.5%) were TargetPrint ER+. The only discordant case showed ER positivity in approximately 85% of tumor nuclei on IHC but was assessed as negative with TargetPrint. This case was PgR+/HER2- by IHC and TargetPrint with a proliferation index (Ki67) of 6%, and was classified as low-risk by both tests, MP and EPclin. For PgR, the positive agreement was 81.6%. Thus, 31 PgR+ cases (77.5%) by IHC were also PgR+ by TargetPrint. Of the 9 discordant cases, 7 tumors were PgR+ by IHC but were classified as PgR- by TargetPrint whereas 2 cases were PgR- by IHC but PgR+ by TargetPrint. Of the 40 HER2- cases by IHC/FISH, 39 (97.5%) were TargetPrint HER2-. The only discordant case was HER2- by both, IHC and FISH, and corresponded to an ER+/PgR+ tumor with a proliferation index (Ki67) of 20%, and was classified as high-risk by both tests, MP and EP.

**Table 5:** Comparison of ER/PgR/HER2 status by IHC/FISH and TargetPrint.

**ER status**		**IHC ER+**	**IHC ER-**		
	TargetPrint ER+	39 (97.5%)	0		
	TargetPrint ER-	1 (2.5%)	0		
**PgR status**		**IHC PgR+**	**IHC PgR-**		
	TargetPrint PgR+	30 (75%)	2 (5%)		
	TargetPrint PgR-	8 (20%)	0		
**HER2 status**		**3+**	**2+/FISH+**	**2+/FISH -**	**Negative 0/1+**
	TargetPrint HER2+	0	0	0	1 (2.5%)
	TargetPrint HER2-	0	0	2 (5%)	37 (92.5%)

ER, estrogen receptor; PgR, progesterone receptor

## Discussion

We compared retrospectively the concordance between EP scores (EP molecular score and EPclin score) and MP scores in 40 ER+/HER2- breast carcinomas. We found a moderate concordance between EP, EPclin and MP-based risk classifications with an overall concordance of 72.5% and 75% between EP molecular score and MP, and EPclin score and MP, respectively.

To the best of our knowledge, this is the first study comparing the concordance in risk classification between the EndoPredict (EP/EPclin) and the MammaPrint tests in a series of ER+/HER2- breast carcinomas. Interestingly, our study shows that despite the discrepancies observed between EP and MP, both tests categorized the patients in the same proportion of cases of high- and low-risk categories (50% each group). These results are similar to those reported in previous studies comparing different gene expression signatures in breast cancer. Comparison of the poor prognosis group of the MP and the intermediate- and high-risk groups from the Oncotype DX recurrence-score (RS) models showed that their sample predictions agreed in 77% of patients with ER+ early-stage breast carcinomas. These analyses suggest that despite very little gene overlap and different algorithms used, the outcome predictions for the majority of these patients would be similar [14]. The Optimal Personalised Treatment of early breast cancer using Multiparameter Analysis preliminary study (OPTIMA prelim) compared risk stratification and subtype classification of five multigene predictor tests (Oncotype DX, Prosigna [PAM50], MammaPrint, MammaTyper, and NexCourse Breast [IHC4-AQUA]) performed in a series of 313 women with ER+/HER- early breast cancer that were randomized to chemotherapy and endocrine therapy or test-directed (chemotherapy if Oncotype DX RS >25) treatment [15]. Strikingly, although the five tests categorized similar numbers of tumors as low- or high-risk categories there was only moderate agreement between tests at the individual patient level. Thus, 60.6% of tumors were assigned to different risk categories by different tests (kappa ranges 0.33–0.60), although 94 (31.1%) showed agreement between four of five tests [15]. Therefore, current multi-parameter tests seem to provide broadly equivalent risk information in ER+/HER2- breast cancers at the population level; however, these tests may provide different risk categorization for the individual patient [15]. Similar findings have been described in a study comparing PAM50 and Oncotype DX [16]. In another study, Varga *et al* reported that the concordance of classification in low- or high-risk between Oncotype DX (combining the intermediate-risk and high-risk groups to one high-risk group) and EP risk score and EPclin score was 76% and 65%, respectively [17].

Although most of the gene signatures currently available provide similar outcome predictions in node-negative patients, significant differences across predictors are present in node-positive disease, and for prediction of late metastasis with improved prognostic power of second generation gene expression tests, such as EP. In this sense, TransATAC, the translational study of the Arimidex, Tamoxifen, Alone or in Combination trial (ATAC), has recently addressed the prognostic value of EP and EPclin for 10-year distant recurrence risk in postmenopausal women with ER+/HER2- breast carcinomas, and compared their prognostic ability with that of the Oncotype DX RS [11]. This study confirmed the independent prognostic ability of EP and EPclin in this cohort of patients. Moreover, EPclin demonstrated a better prognostic ability than RS mainly because of its integration with clinicopathological factors (nodal status and tumor size) but also because of a superior molecular algorithm able to predict late events better than RS, especially in node-positive patients [11]. This led Genomic Health to develop an online Recurrence Score Pathology-Clinical (RSPC) calculator to use in ER+/HER2-/node-negative breast cancer patients; RSPC combines RS with clinicopathological variables, including patient age, tumor size, tumor grade, and planned adjuvant hormonal therapy (tamoxifen or aromatase inhibitor) [18]. RSPC has demonstrated a significantly more prognostic value for distant recurrence compared with RS alone; additionally, it showed better stratification of risk categories in the study population [19].

Multiple factors may contribute to the discrepancies between EP and MP scores. These differences could be partly due to the different main biological motives covered by the genes included in the test algorithms, such as proliferation or ER signaling. Some differences could be explained by the coverage of other motives such as cell adhesion, invasion, or DNA repair, as it has been demonstrated in previous studies comparing EP and Oncotype DX [17]. This heterogeneity may also be attributed partially to the different methodologies that were used to build both classifiers (cDNA microarray and qRT-PCR for MP and EP, respectively), and the heterogeneity in the sample population used to develop the tests. Moreover, each technology has unique normalization methods.

We observed a moderate statistically significant correlation between Ki67 and the EP score, but no correlation between Ki67 and the EPclin score. Similar results have been reported by Varga *et al* who performed a direct comparison of the concordance between Ki67 as a continuous variable and the EP and EPclin scores in a series of 34 ER+ /HER2- breast carcinomas, finding a moderate statistically significant correlation between Ki67 and the EP score (Pearson coefficient 0.55, p<0.0001); no significant correlation was observed between the EPclin score and Ki67 (Pearson coefficient 0.24, p = 0.16) [17]. Using the Ki67 cutoff of 14% Dubsky *et al* divided over 1,000 patients into luminal A and B subtypes and found that the EPclin score can subdivide these patients into two additional groups related to prognosis [12]. Similarly, Filipits *et al* demonstrated that the EP score provided independent prognostic information in a multivariate analysis with conventional clinicopathological factors, including Ki67 (cutoff 11%) [8]. These data suggest that the EP molecular score will likely perform better than Ki67 alone for the prognosis of breast cancer patients, but the performance of the EPclin score *versus* Ki67 is less clear.

TargetPrint, a diagnostic test for the precise molecular readout of ER, PgR, and HER2 mRNA gene expression levels, was additionally analyzed in our series of patients. Our results showed a high concordance between TargetPrint and IHC/FISH for ER (97.5%) and HER2 (97.5%), and moderate concordance for PgR (77.5%). These results are similar to those reported previously, with concordances of approximately 96% for ER and 95% for HER2 [13,20]. Regarding PgR, the concordance for mRNA and IHC analysis has been shown to be approximately 80% in previous studies [21,22]. However, mRNA-derived PgR status is more strongly associated with clinical outcome and, therefore, mRNA could be more reliable for assessing PgR receptor status [23]. The reasons for the discordant results between mRNA readout and IHC assessment of ER, PgR, and HER2 have not been elucidated so far. Suggested possible causes such as intratumoral heterogeneity, have still not been analyzed in a randomized study [20].

The main limitations of this study are related to the moderate sample size which may have limited the conclusions reached in the study. Thus, a further large-scale study including follow-up data is necessary to validate our results.

In conclusion, this study is the first direct comparison of risk stratification between EP/EPclin with MP. Our pilot study shows a limited concordance between EndoPredict and MammaPrint results on individual patients although both tests classified the same proportion of cases into high- and low-risk categories. Recent studies suggest that the addition of clinicopathological variables into these multigene predictor tests, such as EPclin, seems to improve their prognostic ability [24,25]. Discrepant results between tests reflect the fact that the assays are measuring different genes, using different methodology, and highlight the problems of predicting recurrence risk in ER+/HER2- breast cancer patients [26]. Further clinical studies evaluating large patient cohorts including follow-up data are needed to compare both tests.

## Supporting information

S1 Table Detailed description of clinical, pathological, immunohistochemical, and molecular data of the patients included in the study. (XLSX)

## Abbreviations

EP: EndoPredict
ER: estrogen receptor
MP: MammaPrint
PgR: progesterone receptor
